# Supplementary material for: Seasonal dynamics alter taxonomical and functional microbial profiles in Pampa biome soils under natural grasslands
Source: PeerJ. 2018 Jun 13;6:e4991. doi: 10.7717/peerj.4991 (PMC6004115; doi:10.7717/peerj.4991)
Supplement: Table S1 — (1)Extracted with Mehlich-1. (2) Extracted with KCl 1 mol L−1. (3) Walkey & Black method. (4) Extracted with hot water. (5) Pipet method. [file peerj-06-4991-s001.docx]

**Table S1.** Location, soil taxonomy and soil physicochemistry analysis for two grasslands located in Santa Maria and São Gabriel municipalities.

| Characteristics | Sites | |
| --- | --- | --- |
|  | Santa Maria | São Gabriel |
| Latitude (S) | 29⁰45’S | 30°20’S |
| Longitude (W) | 53⁰45’W | 54°15’W |
| Soil Taxonomy | Paleodult | Paleodult |
| Clay (%) ^(5)^ | 16.8 | 17.4 |
| OM^(3)^ | 2.7 | 3.0 |
| Al^3+^ (cmol_c_ dm^-3^) ^(2)^ | 1.3 | 0.3 |
| pH - H2O (1:2,5) | 4.7 | 5.2 |
| P (mg dm^-3^)^(1)^ | 5.0 | 3.7 |
| K (mg dm^-3^) ^(1)^ | 156.0 | 142.0 |
| Ca²⁺ (cmol_c_ dm^-3^) ^(1)^ | 2.2 | 3.8 |
| Mg (cmol_c_ dm^-3^) ^(2)^ | 1.2 | 2.0 |
| Zn (cmol_c_ dm^-3^) ^(1)^ | 1.3 | 2.7 |
| Cu (cmol_c_ dm^-3^) ^(1)^ | 1.2 | 1.3 |
| B (cmol_c_ dm^-3^) ) ^(4)^ | 0.3 | 0.7 |

^(1)^ Extracted with Mehlich-1. ^(2)^ Extracted with KCl 1 mol L^-1^. ^(3)^ Walkey & Black method. ^(4)^ Extracted with hot water. ^(5)^ Pipet method.
