# Supplementary material for: Seasonal dynamics alter taxonomical and functional microbial profiles in Pampa biome soils under natural grasslands
Source: PeerJ. 2018 Jun 13;6:e4991. doi: 10.7717/peerj.4991 (PMC6004115; doi:10.7717/peerj.4991)
Supplement: Table S2 — SM, Santa Maria municipality; SG, São Gabriel municipality. [file peerj-06-4991-s002.docx]

**Table S2.** Sequencing coverage for 16S rRNA (taxa) and mRNA (function) libraries derived from two grasslands located in Santa Maria and São Gabriel municipalities.

| Coverage | | | | | |
| --- | --- | --- | --- | --- | --- |
| Site | Function | Taxa | Site | Function | Taxa |
| Cold Season | | | | | |
| SM1 | 0.881925 | 0.995419 | SG1 | 0.789546 | 0.980991 |
| SM2 | 0.873349 | 0.985071 | SG2 | 0.828687 | 0.978660 |
| SM3 | 0.882867 | 0.980061 | SG3 | 0.825376 | 0.969160 |
| SM4 | 0.789574 | 0.977897 | SG4 | 0.841660 | 0.983947 |
| SM5 | 0.749754 | 0.980536 | SG5 | 0.849093 | 0.986983 |
| SM6 | 0.805065 | 0.971648 | SG6 | 0.872896 | 0.978074 |
| Warm Season | | | | | |
| SM7 | 0.673601 | 0.993183 | SG7 | 0.425860 | 0.978566 |
| SM8 | 0.677982 | 0.990729 | SG8 | 0.703806 | 0.978351 |
| SM9 | 0.683326 | 0.992436 | SG9 | 0.651196 | 0.962931 |
| SM10 | 0.721214 | 0.989151 | SG10 | 0.738881 | 0.962038 |
| SM11 | 0.764248 | 0.994434 | SG11 | 0.883726 | 0.974063 |
| SM12 | 0.700218 | 0.992243 | SG12 | 0.884717 | 0.968737 |

SM = Santa Maria municipality; SG = São Gabriel municipality.
